# A Valuable Contribution to Atrial Fibrillation Ablation in Young Adults: The Role of Long‐Term Follow‐Up and Silent Atrial Fibrillation Monitoring

**DOI:** 10.1002/clc.70182

**Published:** 2025-07-22

**Authors:** Elif Y. Arı, Özden Seçkin, Serkan Ünlü

**Affiliations:** ^1^ Department of Cardiology Gazi University Ankara Turkey

Dear Editor,

We read with great interest the article titled “Clinical Characteristics and Outcomes of Catheter Ablation in Young Patients With Atrial Fibrillation” by Wang et al. [1]. We commend the authors for addressing an important and underexplored area in the management of atrial fibrillation (AF) among young patients.

AF is the most common sustained arrhythmia in adults, and its prevalence has been increasing among younger individuals in recent years. While catheter ablation (CA) has emerged as an effective treatment modality—especially in patients who are symptomatic or refractory to antiarrhythmic drugs—data specific to young populations remain limited. In this context, the study by Wang et al. [1] offers valuable insights into procedural outcomes and recurrence predictors following CA in young adults. Notably, the large sample size and comprehensive reporting of procedural characteristics strengthen the reliability and generalizability of the findings.

However, we would like to highlight some important limitations that deserve consideration. First, the average follow‐up duration reported in the study was only 249 days [1]. While this duration may capture early procedural success, it falls short of assessing long‐term rhythm outcomes, which are particularly relevant in a young population expected to live for decades after the intervention. Late AF recurrences represent a significant clinical issue, especially when sustained sinus rhythm is a primary therapeutic goal in younger patients.

Several studies have demonstrated that AF may recur even many years after apparently successful ablation. For example, Winkle et al. [2] reported a decline in freedom from AF from 68% at 5 years to 48% at 15 years postablation. Similarly, Steinberg et al. [3] and Erhard et al. [4] emphasized that late recurrences are common, and long‐term follow‐up is essential for understanding the true durability of CA. In particular, Erhard et al. showed that arrhythmia recurrence can occur even beyond the first 12 months [4], potentially due to progressive electrical remodeling or emerging triggers. Such late events may necessitate additional interventions or repeat procedures. In light of these findings, Calkins and others have underscored the importance of long‐term surveillance and careful patient selection [5].

Furthermore, as young individuals often experience asymptomatic (silent) AF episodes, relying solely on symptom‐driven follow‐up may lead to underestimation of true recurrence rates. Incorporating prolonged rhythm monitoring techniques—such as extended Holter monitoring or implantable loop recorders—beyond the standard 3‐month blanking period would likely yield a more accurate assessment of long‐term procedural efficacy.

In addition, several reports have highlighted the importance of AF duration before ablation as a strong predictor of success. Patients with shorter disease duration tend to have more favorable atrial substrates and outcomes. Unfortunately, the present study does not provide information on the duration of AF before ablation [1], which limits interpretability. Including this variable in future analyses would enhance the ability to stratify patient risk and tailor treatment strategies accordingly.

In conclusion, we once again congratulate the authors for their valuable contribution to the literature. Nevertheless, we believe that future studies should incorporate extended follow‐up periods and consider preprocedural AF duration as a key determinant of outcomes. Moreover, the use of objective monitoring strategies capable of detecting silent AF episodes will be crucial to accurately evaluate the long‐term success of CA in this unique and increasingly relevant patient population.

## Data Availability

No new data were generated or analyzed in this work.
